# *EGFR* activating mutations detected by different PCR techniques in Caucasian NSCLC patients with CNS metastases: short report

**DOI:** 10.1007/s10585-013-9603-8

**Published:** 2013-07-27

**Authors:** Wojas-Krawczyk Kamila, Skroński Michał, Krawczyk Paweł, Jaguś Paulina, Kucharczyk Tomasz, Jarosz Bożena, Mlak Radosław, Szumiło Justyna, Sawicki Marek, Tomasz Trojanowski, Milanowski Janusz, Chorostowska-Wynimko Joanna

**Affiliations:** 1 Pneumonology, Oncology and Allergology Department, Medical University of Lublin, Jaczewskiego 8, 20-954 Lublin, Poland; 2 Laboratory of Molecular Diagnostics and Immunology, National Institute of Tuberculosis and Lung Disease, Warsaw, Poland; 3 Neurosurgery and Pediatric Neurosurgery Department, Medical University of Lublin, Lublin, Poland; 4 School of Molecular Medicine, Warsaw Medical University, Warsaw, Poland; 5 Clinical Pathomorphology Department, Medical University of Lublin, Lublin, Poland; 6 Thoracic Surgery Department, Medical University of Lublin, Lublin, Poland; 7 Institute of Agricultural Medicine, Lublin, Poland

**Keywords:** *EGFR* mutations, Allele-specific primer PCR, Peptide nucleic acid–locked nucleic acid PCR clamp, NSCLC, Central nervous system metastases

## Abstract

*EGFR* mutation testing has become an essential determination to decide treatment options for NSCLC. The mutation analysis is often conducted in samples with low percentage of tumour cells from primary tumour biopsies. There is very little evidence that samples from metastatic tissues are suitable for *EGFR* testing. We had evaluated the frequency of *EGFR* mutations with three highly sensitive PCR techniques in formalin-fixed, paraffin-embedded samples of 143 NSCLC patients with central nervous system (CNS) metastases. 32 corresponding primary tumours were also examined. We used PCR followed by DNA fragments length analysis (FLA), ASP–PCR and PNA–LNA PCR clamp techniques. We found 9 (6.29 %) *EGFR* gene mutations in CNS samples: 3 (2.1 %) in exon 19 and 6 (4.2 %) in exon 21. The full concordance between CNS metastases and primary tumour samples was observed. PCR followed by DNA–FLA and PNA–LNA PCR clamp were sensitive enough to detect exon 19 deletions. Two mutations in exon 21 were detected by ASP–PCR only, one L858R substitution was detected only by PNA–LNA PCR clamp. With respect to sensitivity, PCR followed by DNA–FLA achieved a level of detection of at least 10 % of mutated DNA for exon 19 deletion, as for ASP–PCR it was at least 5 % of mutated DNA for L858R substitution. Higher sensitivity of 1 % of mutated DNA was achieved by PNA–LNA PCR clamp technique for both mutations. The use of different methodological techniques authenticates the negative result of molecular tests.

## Introduction

Epidermal growth factor receptor (*EGFR*) gene mutations localized within the tyrosine-kinase (TK) domain occur in approximately 10–15 % of Caucasians with lung adenocarcinoma. The majority of activating *EGFR* mutations involve exon 18–21 within the TK domain, including the most frequent short in-frame deletions in exon 19 (mainly delE746-A750) and a specific point mutation in exon 21 affecting codon 858 (L858R) [1]. The effectiveness and reliability of *EGFR* mutation diagnostics in non-small lung cancer (NSCLC) is hindered by numerous methodological challenges, including tumour tissue accessibility, sample quality (low tumour cell content), tumour DNA quality (DNA fragmentation) and the inadequate sensitivity of molecular techniques [2, 3]. Previously, Sanger direct sequencing was considered the gold standard in the molecular diagnosis of *EGFR* mutations. However the success of this method is constrained by strong background signal from the amplified wild-type (wt) *EGFR* allele, requiring a minimum acceptable tumour cell content of 50 % and high quality DNA, thus affecting its practical usefulness in NSCLC clinics [3, 4]. Moreover, recent recommendations from the College of American Pathologists (CAP), International Association for the Study of Lung Cancer (IASLC) and Association for Molecular Pathology (AMP) suggest that methods with higher sensitivity than Sanger sequencing should be applied routinely, since many patients present with low tumour content samples. A significant number of diagnostic samples are derived from biopsy specimens; hence the molecular method must be adequately robust and sensitive to provide reliable results from scant patient material. Consequently, there is an increasing interest in new molecular techniques based on *EGFR* mutant DNA amplification with simultaneous inhibition of wt gene amplification, as well as new generation sequencing [4–7].

To date, the majority of published data assessing such molecular techniques are derived from primary tumour analyses; however, studies assessing the suitability of *EGFR* testing in metastatic tissues are considerably less extensive. The aim of this study was to assess the clinical applicability of three highly sensitive and specific polymerase chain reaction (PCR) techniques, and to perform robust molecular analysis of *EGFR* activating mutations in scant samples of central nervous system (CNS) metastases from Caucasian patients with advanced NSCLC.

## Materials and methods

### Patients’ characteristics

Tumour samples were collected during 2003–2010 from 143 patients with NSCLC who underwent neurosurgery owing to solitary CNS metastases, after obtaining informed, written consent. Patient demographic and clinical characteristics are summarized in Table 1. Except for CNS and lungs, other organs were unaffected by NSCLC. Moreover, only a single metastasis was present in the CNS, enabling tumour excision during neurosurgery. NSCLC not otherwise specified (NOS) was diagnosed in 26.6 % of patients following revision by a second pathologist, mostly owing to the low differentiation of carcinoma. Patient performance status was estimated according to the Zubrod-ECOG-WHO scale. Patients who did not smoke or those with a history of smoking <100 cigarettes were classified as non-smokers, while individuals smoking >100 cigarettes but who had not smoked 5 years prior to the study were considered former smokers. This study was approved by the Ethical Committee of the Medical University in Lublin (KE-0254/131/2011).

**Table 1:** Patients demographics and clinical characteristics

NSCLC patients (*n*=143)	
Median age (mean ± SD) years	59 (59.8 ± 8.9)
Sex	
Female	44 (30.8 %)
Male	99 (69.2 %)
Pathological diagnosis	
Adenocarcinonma	61 (42.6 %)
Squamous cell carcinoma	23 (16.1 %)
Large-cell carcinoma	21 (14.7 %)
NSCLC non other specified (NOS)	38 (26.6 %)
Performance status (PS)	
0	22 (15.4 %)
1	75 (52.4 %)
2	31 (21.7 %)
3	15 (10.5 %)
Smoking history	
Current	77 (53.8 %)
Former	34 (23.8 %)
Never	32 (22.4 %)

### Tumour samples and DNA isolation

Formalin-fixed, paraffin-embedded (FFPE) tissue samples from 143 CNS metastases and 32 FFPE samples from corresponding primary lung tumours were collected. Representative 5–10 μm tissue sections were stained with (H&E) and neoplastic cell content was evaluated by two independent pathologists. Only samples with >10 % cancer cells were considered for further analysis. DNA was isolated from tissue sections using the QIAamp DNA FFPE Tissue Kit (Qiagen, Canada) in accordance with the manufacturer’s instructions.

### *EGFR* gene (exon 19 and 21) analysis by DNA fragment length analysis (DNA–FLA) and allele-specific primer polymerase chain reaction (ASP–PCR)

PCR followed by DNA–FLA and ASP–PCR with CY5 fluorescent-labelled primers (Genomed SA, Warsaw, Poland) was applied to detect short, in-frame deletions in exon 19 and point mutations (L858R) in exon 21 of the *EGFR* gene, respectively. PCR methodology was performed as previously described with further modifications [8]. Analysis was performed with using an ALF Express II sequencer and ALFWin Fragment Analysis software (Amersham Pharmacia, Biosciences, UK). DNA isolated from H1650 and H1975 human NSCLC cell lines, characterized by stable *EGFR* gene mutations in exons 19 and 21 respectively, served as positive controls. DNA isolated from peripheral blood leucocytes of healthy volunteers was used as a negative control.

### *EGFR* gene (exon 19 and 21) analysis using peptide nucleic acid-locked nucleic acid (PNA–LNA) PCR clamp assays

The PNA–LNA PCR clamp assay utilizes a nuclease activity-resistant PNA oligomer that binds to the wt sequence with high affinity, thus inhibiting amplification by PCR. Generic primers were used for amplification of exon 19 and 21 sequences in two reactions for each DNA sample: allele-specific PCR with PNA (+) and control reaction PNA (–). In both reactions, the hydrolysis probes detecting PCR product amplification (“total probe”) and mutation-specific probes were used. Both contain LNA base modifications to improve their binding affinity and specificity. The PNA–LNA PCR clamp real-time assay was performed as previously described with further modifications [9].

All described molecular techniques were used for adequate mutation analysis in all available tumour samples.

### Sensitivity assessment of DNA–FLA, ASP–PCR and PNA–LNA PCR clamp methods

**Fig. 1:**
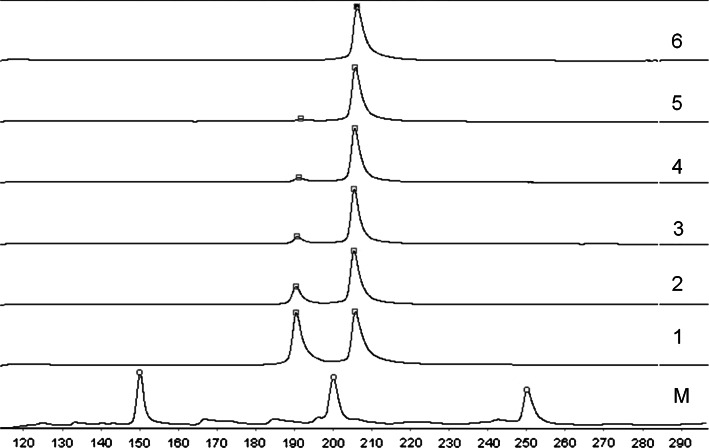
Serial dilutions of mutant DNA (cell line NCI-H1650) with wild-type (wt) DNA (control) to examine the sensitivity of PCR technique for exon 19 mutation. Line: *M* DNA marker, *1* 50 % of wt DNA and 50 % of mutant type DNA; *2* 75 % of wt DNA and 25 % of mutant DNA; *3* 80 % of wt DNA and 20 % of mutant DNA; *4* 90 % of wt DNA and 10 % of mutant DNA; *5* 95 % of wt DNA and 5 % of mutant DNA; *6* 100 % of wt DNA

**Fig. 2:**
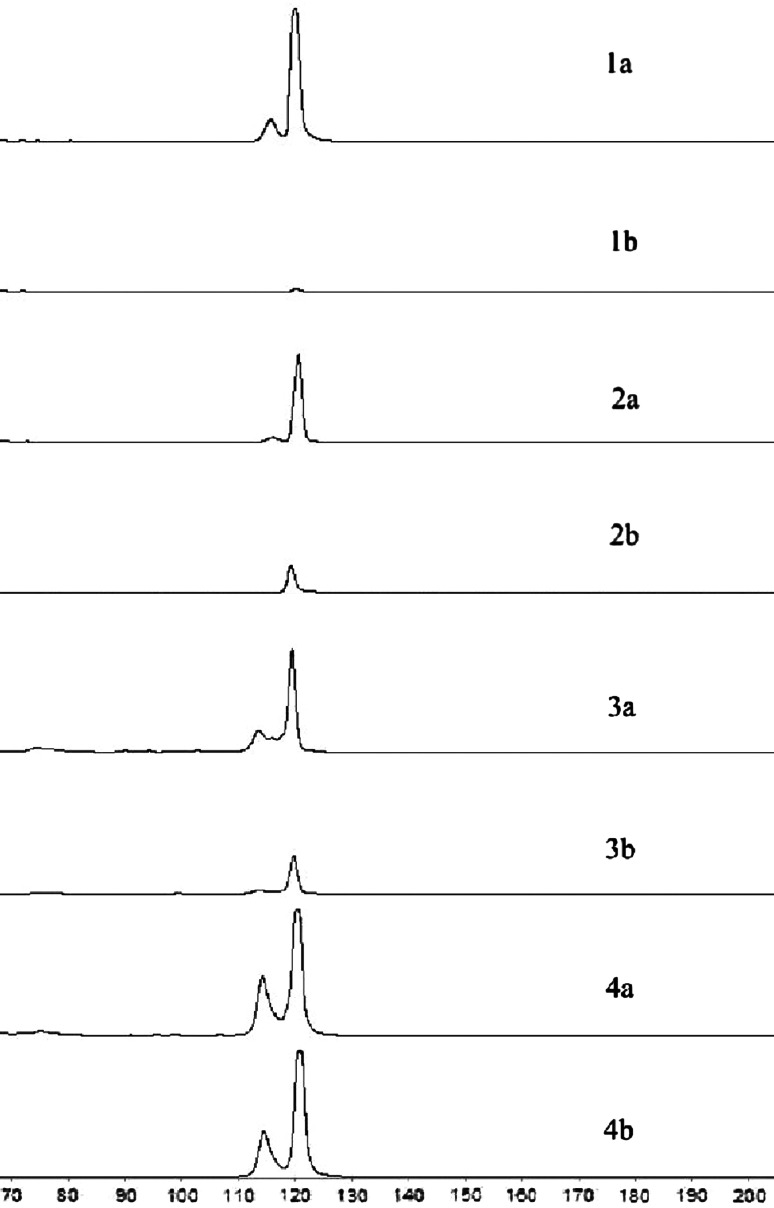
Serial dilutions of mutant DNA (cell line NCI-H1975) in wt DNA (control) to examine the sensitivity of ASP–PCR technique for exon 21 mutation. **a** reaction with primer specific for wt; **b** reaction with primer specific for mutant type. Lines: *1* 98 % of wt and 2 % of mutant DNA. The amplification of mutant type was insufficient for detection. *2* 95 % of wt and 5 % of mutant DNA. *3* 75 % of wt and 25 % of mutant DNA. *4* 50 % of wt and 50 % of mutant DNA

**Fig. 3:**
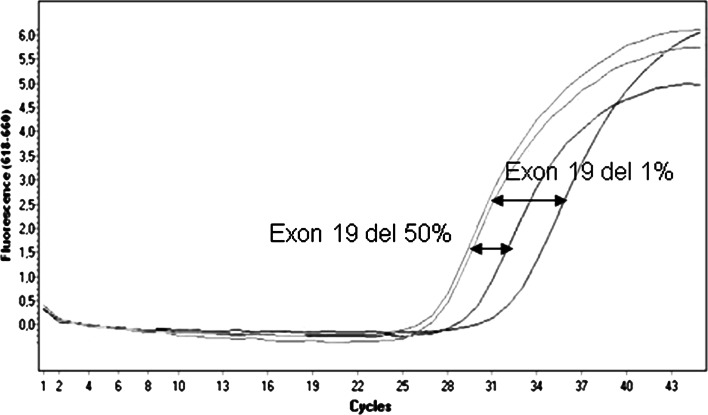
Serial dilutions of mutant genomic DNA (heterozygous for delE746-A750 in *EGFR* gene exon 19) in wt genomic DNA (control) to examine the sensitivity of PNA–LNA PCR clamp technique for *EGFR* exon 19 deletions. For each sample, two reactions were performed – with addition of PNA and without PNA (control). Ct values difference (ΔCt value)for both PNA (+) and PNA (–) reaction was analyzed. 50 % of exon 19 deletion and 50 % of wt DNA (ΔCt = 2.36) and 1 % of exon 19 deletion and 99 % of wt DNA (ΔCt = 5.26)

**Fig. 4:**
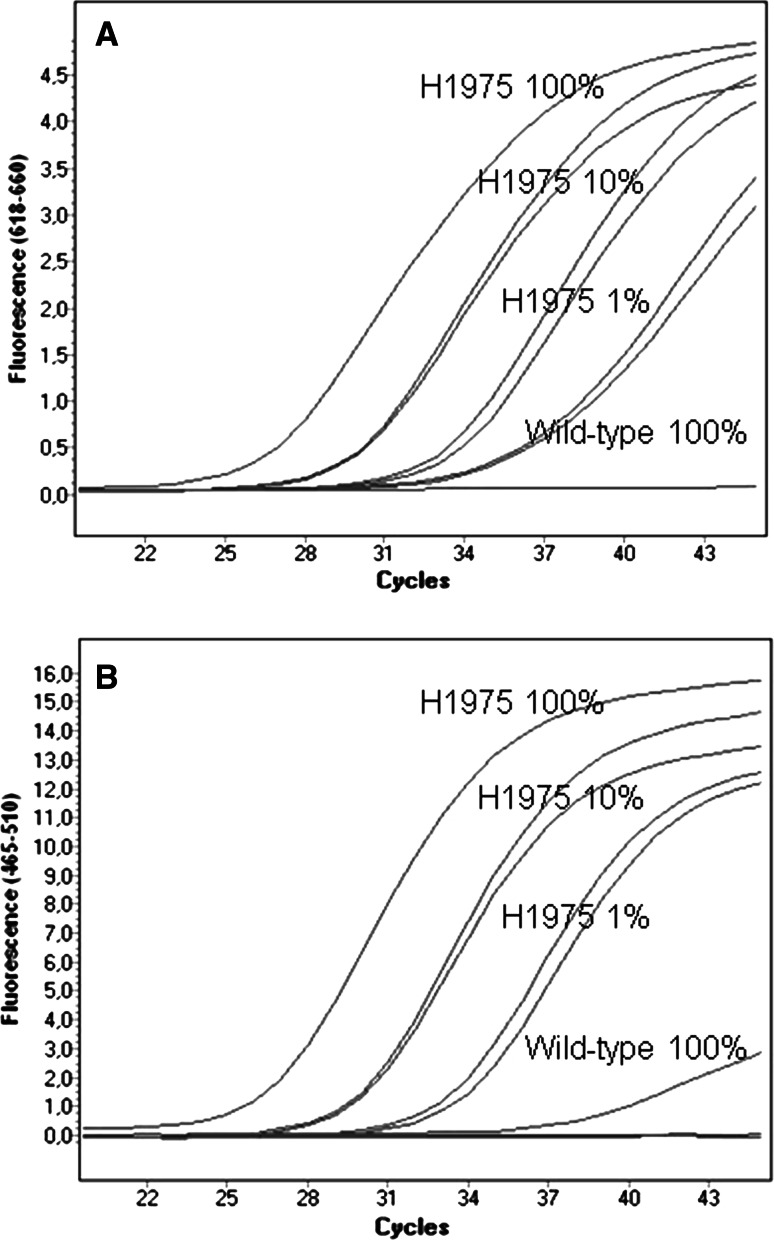
Serial dilutions of mutant genomic DNA (NCI-H1975 cell line heterozygous for L858R mutation in *EGFR* gene exon 21) in wt genomic DNA (control) to examine the sensitivity of PNA–LNA PCR clamp technique for *EGFR* L858R mutation. **a** comparison of PNA–LNA PCR clamp assay results (Cy5-labeled total probes detecting PCR product amplification) for 100, 10 and 1 % dilutions of NCI-H1975 cell line DNA into wt DNA. **b** comparison of PNA–LNA PCR clamp assay results (FAM-labeled probes detecting presence of L858R allele) for 100, 10 and 1 % dilutions of NCI-H1975 cell line DNA into wt DNA

To estimate the sensitivity of the applied techniques, serial dilutions of DNA from NCI-H1650 to NCI-H1975 lung cancer cell lines containing delE747-A750 and L858R mutations were prepared using DNA isolated from healthy donor peripheral blood mononuclear cells (100 % wt and 50, 25, 20, 10, 5, 2 and 1 % mutant DNA by volume). This analysis demonstrated a sensitivity cut-off of 10 % mutant DNA for PCR followed by FLA for delE746-A750 in exon 19 (Fig. 1) and 5 % mutant DNA for detection of the L858R *EGFR* mutation (Fig. 2). A detection cut-off of 1 % mutated DNA was observed for PNA–LNA PCR clamp technique for both mutations (Fig. 3 and 4).

### Statistical analysis

Statistical analysis was performed using Statistica version 8.0. Associations between *EGFR* mutations, patient clinical factors and applied molecular techniques were examined using the Chi square test. *P* values <0.05 were considered statistically significant.

## Results

Tumour specimens from 143 NSCLC patients with CNS metastases were successfully analysed using FLA, ASP–PCR and PNA–LNA PCR clamp molecular techniques. Pre-amplification with nested primers was performed prior to PNA–LNA PCR clamp analysis in 26 % of samples (*n* = 37) owing to poor DNA quality or low quantity of isolated genomic DNA.

Activating mutations of *EGFR* were observed in 6.3 % (9/143) of CNS metastases and included deletion of 15 base-pairs (bp) in exon 19 (delE746-A750) in three cases (2.1 %) and substitution of L858R in exon 21 in six cases (4.2 %). In addition, a rare mutation in exon 21 (A859T) [10] was detected using the PNA–LNA PCR clamp technique, and was subsequently confirmed by direct Sanger sequencing of the PNA-mediated PCR reaction product. However, this mutation was not verified following a repeated PNA–LNA PCR clamp analysis of freshly isolated DNA. Evaluation of primary tumours revealed *EGFR* mutations identical to those identified in corresponding metastases in two patients (one case of — one delE746-A750 in exon 19 and one case of L858R substitution in exon 21). Analysis of primary tumours in the remaining patients with *EGFR* mutation positive CNS metastases was not possible owing to lack of available tissue. Clinical characteristics of patients with activating mutations in the *EGFR* gene are summarized in Table 2.

**Table 2:** Clinical characteristics of patients with activating mutations in *EGFR* gene

No	Type of *EGFR* mutation	Sex	Age	Histopathological diagnosis	Smoking history	Mutation detected in		Techniques
						CNS metastases	Primary tumour	
1	Exon 19 (delE746-A750)	Male	72	Adenocarcinoma	Current smoker	Yes	NA	Detected by PCR followed by FLA and PNA–LNA clamp
2	Exon 19 (delE746-A750)	Female	50	Large-cell carcinoma	Never smoker	Yes	NA	Detected by PCR followed by FLA and PNA–LNA clamp
3	Exon 19 (delE746-A750)	Male	46	Large cell carcinoma	Never smoker	Yes	Yes	Detected by PCR followed by FLA and PNA–LNA clamp
4	Exon 21 (L858R)	Male	56	Adenocarcinoma	Former smoker	Yes	NA	Detected by PNA–LNA clamp and not detected by ASP–PCR
5	Exon 21 (L858R)	Female	73	Adenocarcinoma	Never smoker	Yes	NA	Detected by ASP–PCR and not detected by PNA–LNA clamp
6	Exon 21 (L858R)	Male	55	Adenocarcinoma	Former smoker	Yes	Yes	Detected by ASP–PCR and PNA–LNA clamp
7	Exon 21 (L858R)	Female	61	Adenocarcinoma	Former smoker	Yes	NA	Detected by ASP–PCR and PNA–LNA clamp
8	Exon 21 (L858R)	Female	53	Adenocarcinoma	Never smoker	Yes	NA	Detected by ASP–PCR and not detected by PNA–LNA clamp
9	Exon 21 (L858R)	Female	73	Adenocarcinoma	Never smoker	Yes	NA	Detected by ASP–PCR and PNA–LNA clamp

*NA* not available

*EGFR* gene mutations were observed significantly more frequently in non-smokers compared with smokers (15.62 vs 8.82 % former, 1.29 % current smokers; *p* = 0.014; *λ* ^*2*^ = 6.09); however, there was no significant difference related to gender (11.36 % women vs 4.04 % men, *p* = 0.12; *χ* ^*2*^ = 2.43). Stratification by age did not reveal any significant differences, with a similar percentage of *EGFR* positive patients observed under versus over the age of 60 years (6.67 vs 6.02 %). *EGFR* mutations were predominantly observed in adenocarcinomas (77.8 %); however, two *EGFR* mutations were also detected in large-cell carcinoma metastases (Table 2).

### Conformity of molecular techniques used for detection of *EGFR* mutations

Activating mutations in exon 19 of *EGFR* (delE746-A750) were unequivocally confirmed by PCR followed by FLA and by PNA–LNA PCR clamp techniques in three samples from CNS metastases and in a corresponding sample from a primary tumour. Thus, the observed concordance of these methods for the detection of delE746-A750 was 100 %.

Analysis of the *EGFR* L858R substitution by PCR–ASP and PNA–LNA PCR clamp techniques led to concordant identification of this mutation in 50 % (3/6) of CNS samples and in a corresponding sample from a primary tumour. ASP–PCR identified exon 21 mutations in two additional CNS metastases samples (cases 5 and 8), both of which were negatively screened by PNA–LNA PCR clamp. Conversely, PNA–LNA PCR clamp technique identified the L858R substitution in a CNS metastasis (case 4), not detected by ASP–PCR. *EGFR* L858R mutations were not detected more frequently by ASP–PCR compared with PNA–LNA PCR clamp (*p* = 0.505; *λ* ^*2*^ = 0.44).

## Discussion

In the present study we address two essential questions: first, the incidence of activating *EGFR* mutations in NSCLC metastases to the CNS, and second, the level of concordance between highly sensitive molecular methods routinely used for *EGFR* molecular diagnostics in primary lung tumours.

### Incidence of *EGFR* mutations in NSCLC brain metastases

Brain metastases are one of the most frequent complications of lung cancer and are associated with significant morbidity and mortality [11–13]. Despite this, published data concerning the *EGFR* mutation status of metastatic tumours and corresponding primary lung cancers are limited, particularly in the Caucasian population.

There are several studies evaluating the presence of *EGFR* mutations in CNS lung cancer metastases in the Asian population [14, 15]. Matsumoto et al. [14], examined 21 metastatic brain tumours from 19 NSCLC patients (68 % smokers) and eight samples from corresponding primary lung tumours. *EGFR* mutations were detected in CNS samples from 63 % (12/19) of NSCLC patients, including ten short-in frame deletions in exon 19 and two L858R substitutions in exon 21. In six cases, mutations were identical to those detected in the corresponding primary tumour, while two mutations identified in primary tumours were not consistent with mutations detected in metastatic tumours [14]. In another study, Han et al. [15], observed a 60 % incidence of *EGFR* mutations in NSCLC brain metastases; however, this study was based on a small patient cohort including five NSCLC patients with primary and corresponding brain tumours. In one patient, the L858R substitution present in the primary tumour was not detected in a corresponding metastatic brain sample [15]. In both studies, the frequency of *EGFR* mutations in CNS metastases was typical for the Asian population; however, these observations need to be verified in larger patient cohorts.

In the Caucasian population, the percentage of patients with *EGFR* mutations in NSCLC primary tumours is lower compared with Asian patients (approximately 10–16 %) [2, 16]. A study conducted by Munfus-McCray et al. [17], demonstrated a 40 % incidence of activating *EGFR* mutations in NSCLC brain metastases; however, this study also involved a small cohort (ten examined patients). Grommes et al. also reported a study involving treatment of a very small group of patients (*n* = 9) treated with pulsatile, high-dose erlotinib with CNS metastases and with *EGFR* mutation diagnosed outside of CNS metastases. A partial response of CNS metastases was observed in six patients. Corresponding tissue from the CNS metastases was available for four patients with response after tyrosine kinase inhibitor (TKI)-EGFR therapy diagnosed with *EGFR* mutation matching to those diagnosed outside the CNS metastases (three L858R substitution and one deletion in exon 19) [18].

In the present study, CNS metastases from 143 NSCLC Caucasian patients were examined. We observed activating *EGFR* mutations in 6.29 % of patients. Importantly, complete compliance between *EGFR* mutational status of 32 corresponding primary tumours and brain metastases was observed. Although the calculated incidence of *EGFR* mutations in CNS metastases is lower than previously reported, we are unable to attribute this to technical difficulties since this analysis was performed by two laboratories routinely performing NSCLC molecular diagnostics and involved a large patient cohort. In support of our findings, studies by Lublin and Poznan and Warsaw [19] identified exon 19 and 21 *EGFR* mutations in 10.5 % (*n* = 460) and 9.11 % (*n* = 384) of NSCLC samples, respectively. These results are also compatible with a recent meta-analysis including six randomized studies with a total of 2,797 Caucasian patients with NSCLC (not exclusively lung adenocarcinoma), where the estimated frequency of *EGFR* mutations (exon 19 or 21) was 12.98 %. [20]. The discrepancy between these studies and those reporting higher percentages of *EGFR*-positive patients (>10 %) may be due to the pre-selection of patients based on clinical factors (e.g. histopathological diagnosis, smoking status or qualification for TKI-EGFR therapy). Since our study was not based on a pre-selected patient cohort (with the exception of tumour tissue accessibility), this may account for the lower percentage of *EGFR*-mutated patients. It should be noted, however, that the patient characteristics in our study are not entirely representative of a non-selected NSCLC population in other European countries. The low frequency of patients with adenocarcinoma in our study likely accounts for the low percentage of *EGFR* mutations detected in our group. Alternatively, this may be due to the high percentage of patients within the NSCLC-NOS pathological category (first patients were treated surgically in 2003), as a result of the retrospective nature of the study and since we did not utilize immunohistochemistry antibodies. It should be noted that no *EGFR* mutations were detected in patients with NSCLC-NOS histology, perhaps owing to a lack of adenocarcinoma patients in this group. In addition, our population included a very high percentage of patients with present or past smoking history, characteristic for Polish NSCLC patient populations, which may also account for the low percentage of detectable *EGFR* mutations.

To the best of our knowledge, these are the only publications concerning the frequency of *EGFR* gene mutations in NSCLC CNS metastases. However, a study by Togashii et al. revealed that 50 % (11/22) of patients with *EGFR* gene mutations were also diagnosed with different distant metastases. Moreover, metastasis was diagnosed much less frequently (12 %) in cases of lung adenocarcinoma with wt *EGFR* [21]. Studies by Sun et al. assessed the status of *EGFR* and *KRAS* genes in a cohort of 80 NSCLC patients for whom material from both the primary tumours and the lymph node metastases was available. *EGFR* gene mutations were identified in 21 primary tumours and 26 lymph node metastases, with mutations in primary tumours confirmed in metastases in all cases [22]. Taken together, the role of *EGFR* gene mutations in the occurrence of distant metastases remains controversial.

### Conformity of molecular techniques used for the detection of *EGFR* mutations

To date, molecular diagnostics and lung cancer staging are predominantly performed using histological or cytological material [23–25]. Consequently, the quantity of samples is often limited, with a cancer cell percentage below 50 %, and the DNA yield is correspondingly low. Low cancer cell content is an important issue, since the minimal requirement for accurate detection may be as high as 50 % for Sanger sequencing. Previously, we demonstrated that the median concentration of DNA isolated from intrabronchial forceps biopsy is 38.3 ng/μl [19]. However, commercially available in vitro diagnostic real-time PCR-based tests (CE-IVD) specifically designed for of the detection of *EGFR* activating mutations are not validated to analyse samples with less than 150–800 ng of DNA or 10 % of neoplastic cells [2, 5, 25, 26]. Thus, the development of highly sensitive molecular methods appropriate for more technically demanding samples has become a major focus in lung cancer diagnostics. Techniques based on allele-specific amplification or on the inhibition of wt gene amplification and the simultaneous enhancement of mutated gene amplification have proven particularly useful owing to the high specificity, relative simplicity and cost effectiveness [26–28].

Both allele-specific methods utilized in the present study (PCR followed by DNA fragment analysis and ASP–PCR) demonstrated high detection sensitivity. Previous analyses by Pan et al. utilized an assay to detect exon 19 mutations based on length analysis of fluorescently labelled PCR products. Deletion of exon 19 was readily detected in 6.25 % of DNA from H1650 cells [8]. However, Dahse et al. [28], were able to detect the mutant exon 21 T allele in a mixed sample containing a four fold excess of normal DNA, using an allele-specific PCR for L858R in exon 21.

In our study, the PNA–LNA PCR clamp technique, which inhibits wt gene amplification and simultaneously enhances amplification of the mutated allele, achieved very high sensitivity (1 % of tumour cells for both exons), in accordance with other reports [6, 9, 24]. In an experimental setting, PNA–LNA PCR clamp not only clearly identified mutated alleles intermixed as 1 % of the normal human diploid genome, but also detected one mutant allele in 1,000 diploid human genomes (i.e. 0.1 %) [9]. The reliability of PNA–LNA PCR clamp has been also confirmed in clinical settings, with high sensitivity (97 %) and specificity (100 %) demonstrated in variety of cytological specimens (bronchoscopy samples, sputum, pleural and pericardial effusion) in addition to paraffin-embedded tissues [1, 24, 26]. Accordingly, Yamada et al. [24], demonstrated that the PNA–LNA PCR clamp method allowed positive diagnosis in 33.6 % of 122 cytological samples from Asian NSCLC patients . Studies by Ikeda et al. [26], compared the effectiveness of several highly sensitive PCR methods (ME-PCR, PNA–LNA PCR clamp and PCR invader) to detect *EGFR* mutations in paraffin-embedded tumour sections, frozen cytology specimens obtained by bronchoscopy (washing and brushing) or from malignant pleural effusions. These studies revealed that all methods displayed similar sensitivity, and activating *EGFR* mutations were detected in 28 % (14/50 samples) in a cohort of Asian patients with advanced NSCLC [26].

To our knowledge, this study is the first to compare the consistency of highly sensitive methods in the molecular analysis of intracranial NSCLC metastases. Conflicting results were observed in three of 143 patients evaluated. Since the quantity of specimens available for diagnostic evaluation was generally low, these reported discrepancies were likely due to low material quality. As previously mentioned, pre-amplification of DNA using nested primers was performed owing to DNA fragmentation or low DNA concentration in 37 brain samples. Based on experience with both methods, which are routinely used in our laboratories for NSCLC molecular diagnostics, as well as assumptions based on methodological differences, we hypothesize that PNA–LNA PCR clamp may be more effective in samples with very low tumour cell number, while ASP–PCR may be more sensitive in samples with fragmented DNA.

Our findings and those of other groups, particularly Ikeda et al. [26], provide a rationale for applying at least two molecular techniques in the routine diagnostics of difficult, low-volume or low-quality NSCLC samples, both from primary tumour or metastases. We believe that the use of substantially different methods may allow more consistent results and verification of negative results. Accordingly, discrepant results provided by highly sensitive and specific molecular methods should be rather accepted as true positive rather than false negative results, as exemplified in the Ikeda study. Consequently, we are inclined to recognize the three discrepant results reported in our study as true positives.

The sensitivity of molecular techniques used for the detection of *EGFR* gene mutations is a critical factor in NSCLC diagnosis and subsequent treatment, since the results of these tests may affect qualification for TKI-EGFR-based therapy and the effectiveness of such therapies. Techniques with low sensitivity may lead to disqualification from TKI-EGFR therapy in patients harbouring *EGFR* mutations. Conversely, techniques that are too sensitive may lead to the detection of mutations in rare cell clones within heterogeneous tumours. A study by Kim et al. [29], showed that progression after TKI-EGFR therapy occurs significantly less frequently in patients when *EGFR* mutations are detected by two different techniques (direct sequencing and PNA–LNA PCR clamp), compared with only one method (PNA–LNA PCR clamp, 11.5 vs 22.7 %) .

In conclusion, our analysis of *EGFR* mutations in a homogenous group of 143 Caucasian patients with NSCLC demonstrates that activating *EGFR* mutations are present in 6.29 % of patients, and include exon 19 mutations (2.1 %) and exon 21 mutations (4.2 %). We demonstrate that detection of *EGFR* mutations in NSCLC brain metastases is feasible using highly specific molecular techniques. However, the use of at least two independent molecular methods will ensure a more accurate identification of *EGFR* mutations.

## Abbreviations

ASP: Allele-specific primers
CNS: Central nervous system
EGFR: Epidermal growth factor receptor
FFPE: Formalin-fixed, paraffin-embedded
FLA: Fragments length analysis
PNA–LNA: Peptide nucleic acid–locked nucleic acid

## References

[1] Tanaka T, Matsuoka M, Sutani A, Gemma A, Maemondo M, Inoue A, Okinaga S, Nagashima M, Oizumi S, Uematsu K, Nagai Y, Moriyama G, Miyazawa H, Ikebuchi K, Morita S, Kobayashi K, Hagiwara K (2010). Frequency of and variables associated with the *EGFR* mutation and its subtypes. Int J Cancer.

[2] Angulo B, Conde E, Suárez-Gauthier A, Plaza C, Martínez R, Redondo P, Izquierdo E, Rubio-Viqueira B, Paz-Ares L, Hidalgo M, López-Ríos F (2012). A comparison of *EGFR* mutation testing methods in lung carcinoma: direct sequencing, real-time PCR and immunohistochemistry. PLoS One.

[3] Kamel-Reid S, Chong G, Ionescu DN, Magliocco AM, Spatz A, Tsao M, Weng X, Young S, Zhang T, Soulieres D (2012). *EGFR* tyrosine kinase mutation testing in the treatment of non-small-cell lung cancer. Curr Oncol.

[4] Marchetti A, Normanno N, Pinto C, Taddei GL, Adamo V, Ardizzoni A, Botti G, Bardelli A, Comin C, Crinò L, Fontanini G, Gambacorta M, Marchetti A, Murer B, Normanno N, Nappi O (2010). Recommendations for mutational analysis of *EGFR* in lung carcinoma. Pathologica.

[5] Molina-Vila MA, Bertran-Alamillo J, Reguart N, Taron M, Castellà E, Llatjós M, Costa C, Mayo C, Pradas A, Queralt C (2008). A sensitive method for detecting *EGFR* mutations in non-small cell lung cancer samples with few tumor cells. J Thorac Oncol.

[6] Nakamura T, Sueoka-Aragane N, Iwanaga K, Sato A, Komiya K, Kobayashi N, Hayashi S, Hosomi T, Hirai M, Sueoka E, Kimura S (2012). Application of highly sensitive detection system for epidermal growth factor receptor mutations in plasma DNA. J Thorac Oncol.

[7] Pareek CS, Smoczyński R, Tretyn A (2011). Sequencing technologies and genome sequencing. J Appl Genet.

[8] Pan Q, Pao W, Ladanyi M (2005). Rapid polymerase chain reaction-based detection of epidermal growth factor receptor gene mutations in lung adenocarcinomas. J Mol Diagn.

[9] Nagai Y, Miyazawa H, Huqun, Tanaka T, Udagawa K, Kato M, Fukuyama S, Yokote A, Kobayashi K, Kanazawa M, Hagiwara K (2005). Genetic heterogeneity of the epidermal growth factor receptor in non-small cell lung cancer cell lines revealed by a rapid and sensitive detection system, the peptide nucleic acid-locked nucleic acid PCR clamp. Cancer Res.

[10] Le Maignan L, Mirebeau-Prunier D, Vervueren L, Jeanfaivre T, Urban T, Hureaux J (2011). First case of A859T epidermal growth factor receptor mutation responding to erlotinib. J Thorac Oncol.

[11] Nayak L, Lee EQ, Wen PY (2012). Epidemiology of brain metastases. Curr Oncol Rep.

[12] Porta R, Sánchez-Torres JM, Paz-Ares L, Massutí B, Reguart N, Mayo C, Lianes P, Queralt C, Guillem V, Salinas P, Catot S, Isla D, Pradas A, Gúrpide A, de Castro J, Polo E, Puig T, Tarón M, Colomer R, Rosell R (2011). Brain metastases from lung cancer responding to erlotinib: the importance of *EGFR* mutation. Eur Respir J.

[13] Jamal-Hanjani M, Spicer J (2012). Epidermal growth factor receptor tyrosine kinase inhibitors in the treatment of epidermal growth factor receptor-mutant non-small cell lung cancer metastatic to the brain. Clin Cancer Res.

[14] Matsumoto S, Takahashi K, Iwakawa R, Matsuno Y, Nakanishi Y, Kohno T, Shimizu E, Yokota J (2006). Frequent *EGFR* mutations in brain metastases of lung adenocarcinoma. Int J Cancer.

[15] Han HS, Eom DW, Kim JH, Kim KH, Shin HM, An JY, Lee KM, Choe KH, Lee KH, Kim ST, Koo JH, Lee HC, Lee OJ (2011). *EGFR* mutation status in primary lung adenocarcinomas and corresponding metastatic lesions: discordance in pleural metastases. Clin Lung Cancer.

[16] Rosell R, Moran T, Queralt C, Porta R, Cardenal F, Camps C, Majem M, Lopez-Vivanco G, Isla D, Provencio M, Insa A, Massuti B, Gonzalez-Larriba JL, Paz-Ares L, Bover I, Garcia-Campelo R, Moreno MA, Catot S, Rolfo C, Reguart N, Palmero R, Sánchez JM, Bastus R, Mayo C, Bertran-Alamillo J, Molina MA, Sanchez JJ, Taron M, Spanish Lung Cancer Group (2009). Screening for epidermal growth factor receptor mutations in lung cancer. N Engl J Med.

[17] Munfus-McCray D, Harada S, Adams C, Askin F, Clark D, Gabrielson E, Li QK (2011). *EGFR* and *KRAS* mutations in metastatic lung adenocarcinomas. Hum Pathol.

[18] Grommes C, Oxnard GR, Kris MG, Miller VA, Pao W, Holodny AI, Clarke JL, Lassman AB (2011). “Pulsatile” high-dose weekly erlotinib for CNS metastases from *EGFR* mutant non-small cell lung cancer. Neuro Oncol.

[19] Krawczyk P, Ramlau R, Powrózek T, Wojas-Krawczyk K, Sura S, Jarosz B, Walczyna B, Pankowski J, Szumiło J, Dyszkiewicz W, Woźniak A, Milanowski J (2012). The detection of *EGFR* mutations in patients with non- small cell lung cancer in selected center in Poland involved in the molecular diagnostics. Kardiochirurgia i Torakochirurgia Polska.

[20] Petrelli F, Borgonovo K, Cabiddu M, Barni S (2012). Efficacy of EGFR tyrosine kinase inhibitors in patients with *EGFR*-mutated non-small-cell lung cancer: a meta-analysis of 13 randomized trials. Clin Lung Cancer.

[21] Togashi Y, Masago K, Kubo T, Sakamori Y, Kim YH, Hatachi Y, Fukuhara A, Mio T, Togashi K, Mishima M (2011). Association of diffuse, random pulmonary metastases, including miliary metastases, with epidermal growth factor receptor mutations in lung adenocarcinoma. Cancer.

[22] Sun L, Zhang Q, Luan H (2011). Comparison of KRAS and EGFR gene status between primary non-small cell lung cancer and local lymph node metastases: implications for clinical practice. J Exp Clin Cancer Res.

[23] van Eijk R, Licht J, Schrumpf M, Talebian Yazdi M, Ruano D, Forte GI, Nederlof PM, Veselic M, Rabe KF, Annema JT, Smit V, Morreau H, van Wezel T (2011). Rapid *KRAS*, *EGFR*, *BRAF* and *PIK3CA* mutation analysis of fine needle aspirates from non-small-cell lung cancer using allele-specific qPCR. PLoS ONE.

[24] Yamada N, Oizumi S, Asahina H, Shinagawa N, Kikuchi E, Kikuchi J, Sakakibara-Konishi J, Tanaka T, Kobayashi K, Hagiwara K, Nishimura M (2012). The peptide nucleic acid-locked nucleic acid polymerase chain reaction clamp-based test for epidermal growth factor receptor mutations in bronchoscopic cytological specimens of non-small cell lung cancer. Oncol.

[25] Lopez-Rios F, Angulo B, Gomez B, Mair D, Martinez R, Conde E, Shieh F, Tsai J, Current R, Lawrence HJ, Gonzales de Castro D (2012) Comparison of molecular testing methods for the detection of *EGFR* mutations in formalin-fixed paraffin-embedded tissue (FFPET) specimens of non-small cell lung cancer (NSCLC). European Lung Cancer Conference Geneva, abs. 270 10.1136/jclinpath-2012-201240 PMC3632986 23386666

[26] Ikeda T, Nakamura Y, Yamaguchi H, Tomonaga N, Doi S, Nakatomi K, Iida T, Motoshima K, Mizoguchi K, Nagayasu T, Tsukamoto K, Kohno S (2012). Direct comparison of 3 PCR methods in detecting *EGFR* mutations in patients with advanced non-small-cell lung cancer. Clin Lung Cancer.

[27] Asano H, Toyooka S, Tokumo M, Ichimura K, Aoe K, Ito S, Tsukuda K, Ouchida M, Aoe M, Katayama H, Hiraki A, Sugi K, Kiura K, Date H, Shimizu N (2006). Detection of *EGFR* gene mutation in lung cancer by mutant-enriched polymerase chain reaction assay. Clin Cancer Res.

[28] Dahse R, Berndt A, Kosmehl H (2006). PCR-based testing for therapy-related *EGFR* mutations in patients with non-small cell lung cancer. Anticancer Res.

[29] Kim HJ, Lee KY, Kim YC, Kim KS, Lee SY, Jang TW, Lee MK, Shin KC, Lee GH, Lee JC, Lee JE, Kim SY (2012). Detection and comparison of peptide nucleic acid-mediated real-time polymerase chain reaction clamping and direct gene sequencing for epidermal growth factor receptor mutations in patients with non-small cell lung cancer. Lung Cancer.

